# Prevalence of vision impairment in Rwanda: a hospital-based study

**DOI:** 10.3389/fmed.2025.1701330

**Published:** 2025-11-17

**Authors:** Benedict Ayobi, Rekha Hansraj, Nishanee Rampersad, Gerard Urimubenshi

**Affiliations:** 1 Discipline of Optometry, College of Health Sciences, University of KwaZulu-Natal, Durban, South Africa; 2 Department of Ophthalmology, College of Medicine and Health Sciences, University of Rwanda, Kigali, Rwanda; 3 Department of Physiotherapy, College of Medicine and Health Sciences, University of Rwanda, Kigali, Rwanda

**Keywords:** vision impairment, blindness, cataracts, refractive error, Rwanda

## Abstract

**Background:**

Vision impairment (VI) is a global health challenge with its impact cutting across ages, gender, and all domains of life. A shift in global demographics due to increasing life expectancy is expected to increase the burden of VI.

**Aim:**

This study sought to determine the prevalence of VI in the general population among selected public hospitals in Kigali and southern Rwanda.

**Methods:**

This retrospective hospital-based study used five public hospitals from Kigali and Southern Rwanda. Patient files from 1st January 2018 to 31st December 2023 were sampled, and files with VI according to defined criteria were selected, and data were collected. Data were analyzed using descriptive and inferential statistics.

**Results:**

Most patients who presented to the hospitals were female (60.7%). The prevalence of VI ranged between 8.1 and 18.2%. VI was more prevalent in females than in males (18.0% vs. 13.7%). Additionally, VI was most prevalent among the elderly (29.1%). The leading causes of VI were diseases of the lens and normal globe diseases. The risk of VI was significantly higher in males (AOR: 1.25; 95% CI: 1.16–1.34), the elderly (AOR: 1.57; 95% CI: 1.40–1.76), and those with diseases of the lens (AOR: 2.17; 95% CI: 1.88–2.51).

**Conclusion:**

The burden of VI in Rwanda is expected to increase due to a growing youthful population. Sustained efforts, including improving human resources and addressing the unmet demands of cataract and refractive error, are critical to avert the impending public health challenge.

## Introduction

The impact of vision impairment (VI) has been extensively studied, with the effects noted to have far-reaching consequences that transcend ages, social domains, cultures, and all facets of life. The presence of VI in adults restricts movement, directly affecting ambulation, and leads to dependence and subsequent challenges with activities of daily living (1). Additionally, the quality of life diminishes due to challenges with everyday tasks, such as cooking, eating, and shopping, which VI affects (1, 2). In children, VI may negatively impact their academic performance in school due to their inability to engage in academic tasks, as well as their social well-being. Children of school-going age with VI are sometimes perceived as dull and unintelligent, often due to their limited participation in school activities (1, 3).

Economically, VI places an overwhelming burden on the community’s finances, affected individuals, and their caregivers. In the United States of America alone, it is estimated that 98.7 billion dollars is spent annually on direct medical costs associated with VI (4). The lack of independence associated with VI often results in a loss of productivity for individuals with VI and their caregivers (5). Furthermore, it has been documented that VI increases the risk of mortality (1). Reduced independence, coupled with limited employment opportunities for persons with VI, leads to an increased risk of suicide among patients with VI (6). Additionally, reduced mobility due to VI can lead to falls and serious injuries (7, 8). Consequently, VI affects all domains of life of the individual and their community.

VI is a global health concern with a prevalence of 4.34% as of 2020 (9). This prevalence is expected to double over the next three decades due to a gradual shift in the world’s demographics towards an older population, resulting from increasing life expectancy (9, 10). Previous studies have established that there is an unequal distribution of VI worldwide (11, 12). It is estimated that the prevalence of VI is about four times higher in developing countries, especially in Africa, than in developed countries (1) with a recent systematic review (13) reporting the prevalence of VI in East Africa to be between 1.6 and 42.1%. Lack of access to eye care services, lower socioeconomic status, and a paucity of data from developing countries are some of the reasons that may contribute to the disparity in the reported prevalence of VI. Additionally, the unavailability of suitably trained personnel, stigmatisation, and discrimination against people with VI, as well as the cost of accessing eye care services, have contributed to the higher prevalence of VI in developing countries (1, 14, 15).

Over the years, global efforts have intensified to address the prevalence of VI. One such effort was the VISION 2020 initiative by the World Health Organization (WHO) and the International Agency for Preventing Blindness (IAPB), which aimed to reduce avoidable VI and blindness (16). Among other targets, VISION 2020 promoted the training of eye care professionals and made eye care services accessible and affordable (17). After this initiative, Rwanda, an East African country, also introduced country-specific policies to address the prevalence of VI. Together with donor partners, Rwanda introduced programs to address human resource development (training eye care cadres) and infrastructure challenges, conduct disease burden studies, and provide low-cost and/or free spectacles to those in need (18).

Early identification and treatment of the causes of VI cannot be overlooked if the burden of disease and its impact on society, the family, and the individual are to be addressed. A literature search indicates that no study has been conducted on the prevalence of VI in Rwanda in the general population. Previous studies in Rwanda have reported the prevalence of VI between 1.6 and 5.3% (19–22). Two studies reported on the prevalence of VI among persons aged 50 and above, while two studies reported on the prevalence of VI in children (aged 18 years and below). Though the reported prevalence of VI may have been low, the previous studies did not account for other age groups in the community. The percentage of persons 50 years and above in Rwanda is estimated to be 11.88%, while persons below 50 make up the most significant proportion of the population at 88.12% (23). The burden of VI in developing countries, including Rwanda, is estimated to be higher than previously reported. Therefore, early interventions, such as ascertaining the prevalence and burden of disease by early diagnosis, documentation, and treatment of the causes of VI, will help plan and implement strategies to address the prevalence of VI in the general population (12). Therefore, this study sought to determine the prevalence of VI in the general population in Kigali and Southern Rwanda by assessing the presentation of VI at selected hospitals in Kigali and Southern Rwanda.

## Methods

### Study design, population, and sampling

The study was a retrospective, hospital-based, cross-sectional study that involved five public hospitals that provide eye care services. The study employed a mixed sampling method where four hospitals were randomly selected by drawing from a hat to represent the Kigali administrative district, and one hospital was purposively selected from the southern province of Rwanda. One hospital was purposively selected from the Southern Province because it is the major hospital that provides full scope of eye care services in the Province of Rwanda. Furthermore, the only other public referral hospital had recently transitioned from analog to electronic data management, resulting in loss of data, including that required for the study period. Public hospitals were chosen for this study for two reasons. Firstly, more than 86% of the Rwandan population is registered with the government’s medical health insurance (Mutuelle de santé), which grants access to healthcare in public hospitals only (24). Secondly, private hospitals declined to grant access to patients’ medical records during preliminary discussions with them. The study included all medical records of patient visits to the eye clinics of the selected public hospitals from January 1, 2018, to December 31, 2023. All patient files from patient visits for a selected year were sampled. In total, 123,211 patient files were sampled. Then, patient medical records identified as VI, as per the criteria detailed below, were selected, and data were extracted. To avoid double entries, the researchers considered patient data from each patient’s first visit for a particular year. If the patient presented with a pathology (such as a corneal ulcer or trauma) that required frequent review, the researchers considered the details of the last visit. Subsequent reports of the same patient visit within the same year were excluded. Patients referred between any of the hospitals involved in this study were also excluded from the sample.

### Data collection procedure

The principal researcher conducted a pilot study from January 1st to January 31st, 2024, using data from a hospital not part of the selected study sites to validate the data extraction sheet and the study design. Based on the pilot study, the researchers decided to categorize the causes of VI according to the anatomical site of the disease that led to VI. In addition, certain data fields, including occupation, residence, and best correct VA, were removed from the data extraction sheet as they were not consistently available. As reported elsewhere (25, 26), the causes of VI were grouped into the following: whole globe causes (glaucoma, phthisis bulbi, microphthalmos and anophthalmus); cornea (staphyloma, corneal scar, corneal opacity, keratoconus); lens (cataract, aphakia, pseudophakia); retina (atrophy, dystrophy, retinoblastoma, retinitis pigmentosa, diabetic and hypertensive retinopathies, macular degeneration, macula hole, retinopathy of prematurity, retinal detachment); optic nerve (optic atrophy); normal globe (refractive error, nystagmus, amblyopia and cortical blindness) and uvea (uveitis). This was to address the heterogeneity in the diagnosis from various facilities. If a participant was diagnosed with more than one disease as the cause of VI, that patient’s principal cause of VI was selected as the disease that is more amenable to treatment, or, if not treatable, the one that is more amenable to prevention (27). Data from the pilot study were excluded from the research sample. Data collection involved the use of a validated data extraction sheet. Socio-demographic information, such as age and gender, and clinical profiles including presenting visual acuity (VA), diagnosis, and treatment administered, were recorded.

### Definitions

Vision Impairment (VI) was classified using the International Classification of Diseases 11 (28). No VI was defined as presenting visual acuity (PVA) equal to or better than 6/12 in the better seeing eye. Presenting VA worse than 6/12 but better than 6/18 was classified as mild VI, PVA worse than 6/18 but better than or equal to 6/60 in the better-seeing eye was classified as moderate VI, and PVA worse than 6/60 but better than 3/60 was classified as severe VI. Blindness was defined as PVA worse than 3/60 in the better-seeing eye, with optical correction, if any (Appendix 1) (28). Refractive error was classified as myopia if the spherical power was ≤ − 0.50 Dioptre Sphere (DS) and hyperopia if it was ≥ + 0.50 DS. Astigmatism was considered significant when it was >0.50 Dioptre cylinder (15, 29). For ease of analysis, participants were grouped into four age ranges: children (<18 years), youth (18–35 years), adults (36–59 years), and the elderly (>60 years).

### Data management and analysis

After cleaning and ensuring the completeness of the data, the researcher coded and entered the data into the Statistical Package for Social Sciences (version 30; SPSS Inc., Chicago, IL, USA) for analysis. Data were summarized using descriptive statistics, including frequencies and measures of central tendency. The results are presented in the form of tables and graphs. Multivariate logistic regression was used to identify the relationship between VI and associated demographic factors. A *p*-value of less than 0.05 was considered statistically significant.

## Results

### Prevalence and distribution of VI

The prevalence and distribution of VI is detailed in Table 1. From 2018 to 2023, patient numbers at the selected hospitals increased steadily from 13,459 to 24,176, with females consistently representing about 60% of the hospital attendance. The elderly (≥60 years) and adults (36–59 years) formed the largest groups, while children showed a notable rise in 2023. Vision impairment prevalence grew from 8.1% in 2018 to a peak of 18.2% in 2022 before declining marginally to 14.5% in 2023; with moderate VI being the most common VI. Females generally had higher VI prevalence than males, though male cases increased markedly in 2022. Across all years, the elderly had the greatest burden of VI, accounting for over 60% of cases, followed by adults, while children and youth contributed smaller proportions.

**Table 1:** Prevalence and clinical characteristics of VI among sampled patient files.

Year of visit	2018	2019	2020	2021	2022	2023
Total number of patients	13,459	18,086	20,041	23,600	23,849	24,176
Gender (number/%)						
Male	5,357 (39.8%)	7,098 (39.2%)	7,682 (38.3%)	9,557 (40.5%)	9,039 (37.9%)	9,709 (40.2%)
Female	8,102 (60.2%)	10,988 (60.8%)	12,359 (61.7%)	14,043 (59.5%)	14,810 (62.1%)	14,467 (59.8%)
Age (years)						
Children (<18)	2060 (15.3%)	2,581 (14.3%)	3,430 (17.1%)	3,722 (15.8%)	3,859 (16.2%)	4,940 (20.4%)
Youth (18–35)	3,134 (23.3%)	4,047 (22.4%)	5,274 (26.3%)	5,339 (22.6%)	4,958 (20.8%)	5,495 (22.7%)
Adults (36–59)	3,769 (28.0%)	4,867 (26.9%)	5,895 (29.4%)	6,729 (28.5%)	6,811 (28.6%)	6,023 (24.9%)
Elderly (≥60)	4,496 (33.4%)	6,591 (36.4%)	5,442 (27.3%)	7,810 (33.1%)	8,221 (34.5%)	7,718 (31.9%)
Presence of VI						
Yes	1,094 (8.1%)	2,805 (15.5%)	3,020 (15.1%)	4,209 (17.8%)	4,332 (18.2%)	3,513 (14.5%)
No	12,365 (91.9%)	15,281 (84.5%)	17,021 (84.9%)	19,391 (82.2%)	19,517 (81.8%)	20,663 (85.5%)
Classification of VI						
Mild VI	224 (1.7%)	340 (1.9%)	711 (3.5%)	1,174 (5.0%)	1,002 (4.2%)	652 (2.7%)
Moderate VI	369 (2.7%)	962 (5.3%)	1,076 (5.4%)	1,425 (6.0%)	1,471 (6.2%)	1,476 (6.1%)
Severe VI	235 (1.7%)	762 (4.2%)	583 (2.9%)	740 (3.1%)	883 (3.7%)	715 (3.0%)
Blind VI	266 (2.0%)	741 (4.1%)	650 (3.2%)	870 (3.7%)	976 (4.1%)	670 (2.8%)
No VI	12,365 (91.9%)	15,281 (84.5%)	17,021 (84.9%)	19,391 (82.2%)	19,517 (81.8%)	20,663 (85.5%)
VI among genders						
Male	525 (9.8%)	1,380 (19.4%)	1,489 (19.4%)	1986 (20.8%)	1,625 (18.2%)	1720 (17.7%)
Female	569 (7.0%)	1,425 (13.0%)	1,531 (12.4%)	2,223 (15.8%)	2,707 (18.3%)	1793 (12.4%)
VI among age groups						
Children (<18)	125 (11.4%)	158 (5.6%)	340 (11.3%)	312 (7.4%)	264 (6.1%)	459 (13.1%)
Youth (18–35)	157 (14.4%)	212 (7.6%)	385 (12.7%)	504 (12.0%)	398 (9.2%)	397 (11.3%)
Adults (36–69)	193 (17.6%)	472 (16.8%)	573 (19%)	815 (19.4%)	873 (20.2%)	613 (17.4%)
Elderly (≥60)	619 (56.6%)	1963 (70.0%)	1722 (57.0%)	2,578 (61.2%)	2,797 (64.5%)	2044 (58.2%)

### Trend of presentation of VI in selected hospitals

**Figure 1:**
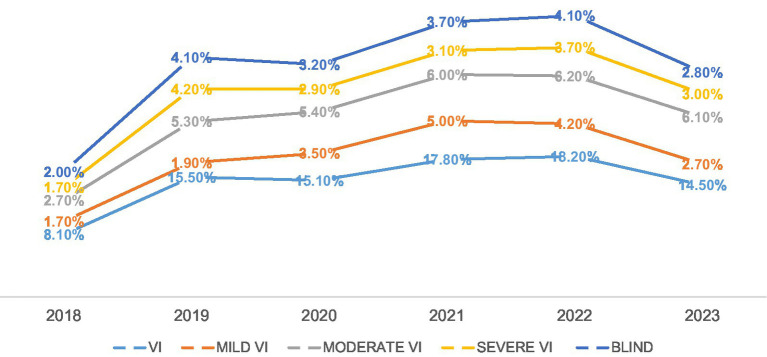
Trend of presentation of VI over the study period.

The trend of VI presented at selected hospitals over the 6 years of this study showed a relatively stable prevalence of VI after a steep rise, followed by a decline (Figure 1). In 2018, the prevalence of VI was 8.1%. There was a steep increase in 2019 to 15.5%. The prevalence of VI continued to increase in 2021 (17.8%) after a marginal decline in 2020 (15.1%). The highest prevalence of VI was recorded in 2022 (18.2%). Then, after, the reported prevalence of VI declined to 14.5% (Figure 1).

### Causes of VI

**Figure 2:**
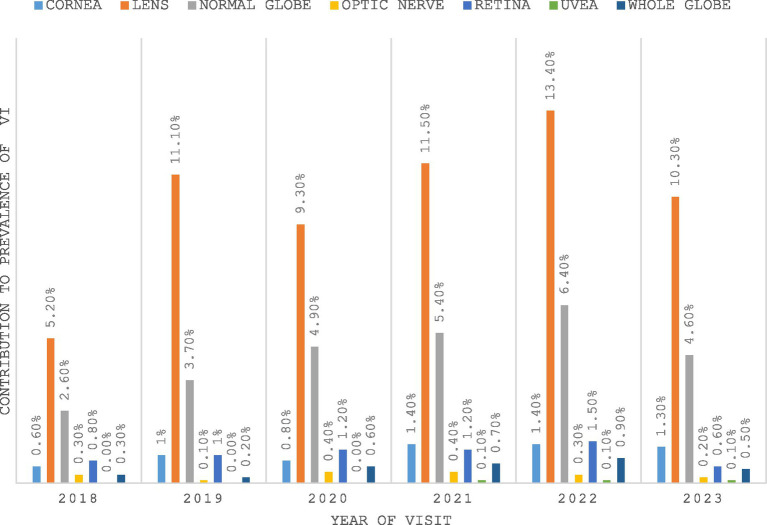
Causes of VI as noted in the sampled files.

The two leading causes of VI were diseases that affected the lens, followed by those that affected the normal globe (Figure 2). Over the study period, diseases of the lens consistently contributed most to the prevalence of VI, with the highest contribution recorded in 2022 (13.4%). This was followed by diseases affecting the normal globe, including refractive errors. The prevalence of normal globe diseases increased steadily from 2018 (2.6%) to 2022 (6.4%), then marginally decreased in 2023 (4.6%).

Overall, the causes of VI increased with age for all causes except diseases of the lens and retina, which were predominantly prevalent among the elderly. Diseases of the lens and retina were the leading causes of VI among patients aged 60 years or older, accounting for 73.6 and 53.4% of cases, respectively. For all other causes, the trend increased gradually until it peaked in the elderly group (Table 2).

**Table 2:** Number and proportion of cases for each cause of VI, stratified by age group.

Age category	<18 years	18–35 years	36–59 years	≥60 years
Cause of VI				
Cornea	175 (15.8%)	336 (30.4%)	175 (15.8%)	419 (37.9%)
Lens	753 (6.4%)	545 (4.7%)	1795 (15.3%)	8,622 (73.6%)
Normal globe	565 (13.5%)	893 (21.2%)	983 (23.4%)	1757 (41.9%)
Optic nerve	32 (10.6%)	87 (28.7%)	89 (29.3%)	95 (31.4%)
Retina	58 (5.7%)	92 (9.1%)	321 (31.8%)	540 (53.4%)
Uvea	2 (7.4%)	8 (29.6%)	14 (51.9%)	3 (11.1%)
Whole globe	36 (8.1%)	59 (13.2%)	120 (26.9%)	231 (51.8%)

Numbers are raw counts of individual cases, and percentages are proportions of causes of VI.

### Factors associated with VI

A binary logistic regression analysis (Table 3) showed that being male (OR: 1.38; 95% CI: 1.34–1.43), aged 36–59 years (OR: 1.32; 95% CI: 1.24–1.41), and/or aged ≥60 years (OR: 4.67; 95% CI: 4.44–4.96) had a significantly increased risk of VI. Regarding the causes of VI, diseases of the lens (OR: 2.66; 95% CI: 2.31–3.05) and optic nerve diseases (OR: 1.79; 95% CI: 1.29–2.49) were significantly associated with increased risk of VI. After adjusting for age and gender, males had higher odds of VI (AOR: 1.25; 95% CI: 1.16–1.34), adults (AOR: 1.14; 95% CI: 1.00–1.30), and the elderly had nearly two times the odds of VI (AOR: 1.57; 95% CI: 1.40–1.76). Regarding the causes of VI, diseases of the lens (AOR: 2.17; 95% CI: 1.88–2.51) and optic nerve diseases (AOR: 1.76; 95% CI: 1.26–2.45) were significantly associated with increased risk of VI.

**Table 3:** Logistic regression for vision impairment.

Factors associated with VI	Unadjusted odds			Adjusted odds for age and gender		
	OR	95% CI	*P*	AOR	95% CI	*P*
Gender						
Female	Ref	–	–	Ref	–	–
Male	1.38	1.34–1.43	<0.001	1.25	1.16–1.34	<0.001
Age (years)			<0.001			<0.001
<18	ref			Ref	–	–
18–35	0.90	0.84–0.96	0.001	0.87	0.77–1.00	0.043
36–59	1.32	1.24–1.41	<0.001	1.14	1.00–1.30	0.036
≥60	4.67	4.44–4.96	<0.001	1.57	1.40–1.76	<0.001
Site of abnormality			<0.001			<0.001
Cornea	Ref	–	–	Ref	–	–
Lens	2.66	2.31–3.05	<0.001	2.17	1.88–2.51	<0.001
Normal globe	0.71	0.62–0.81	<0.001	0.67	0.58–0.77	<0.001
Optic nerve	1.79	1.29–2.49	<0.001	1.76	1.26–2.45	<0.001
Retina	0.95	0.79–1.13	0.561	0.84	0.70–1.00	0.062
Uvea	0.16	0.10–0.27	<0.001	0.18	0.11–0.29	<0.001
Whole globe	0.48	0.40–0.59	<0.001	0.43	0.35–0.53	<0.001

## Discussion

With increasing life expectancy, the prevalence of VI is a significant concern for healthcare personnel and policymakers, especially in Africa. Vision impairment has far-reaching consequences, affecting individuals with VI, caregivers, and society. The prevalence of VI is estimated to be higher than previously reported; therefore, it is imperative that adequate steps are taken to address the burden of VI in the community. Additionally, identifying the leading causes of VI will aid policy direction on the specific actions and strategies needed to manage the burden of VI in the community. This study, therefore, sought to determine the prevalence, causes, and associated risk factors of VI among patients presenting to public hospitals in Kigali and Southern Rwanda. The prevalence of VI among the general population in Rwanda ranged between 8.1 and 18.2%. A notable observation is the sharp increase in the prevalence of VI between 2018 and 2019 (8.1 to 15.5%). This may be attributed to the increase in utilization of eye care services following the implementation of the eye care Performance-Based Financing model (ePBF). The Ministry of Health in Rwanda adopted the PBF model in 2019 to increase access (quantity) and utilization (quality) of eye care services at the primary care level by maximizing scarce resources. This initiative saw a sustained rise (fourfold increase) in the number of patients who sought general and eye consultations between 2017 and 2022 (30). The ePBF was adapted in response to the Rwanda National Strategic Policy for eye care in 2018. The Strategic Plan for Eye Care aimed to improve access to eye care and reduce avoidable blindness (30–32). Although the prevalence of VI in this study is concerning, it is lower than that found in other hospital-based studies in the subregion. Current and comparable hospital-based studies reported the prevalence of VI in Sudan to be 28.5% (33), Ethiopia (28.6%) (34), Ghana (28.4%) (35), Nigeria (33.97%) (36) and South Africa (61.5%) (37). The significant difference between the reported prevalence in this study and that in previous studies may be attributed to the socioeconomic status of the study participants and the sampling techniques employed in each study. To demonstrate this, the study in South Africa was conducted in Limpopo, an economically less endowed province. The authors alluded that higher poverty rates and lack of access to ophthalmology services may have contributed to the higher prevalence in that study (37). A marginal decrease in the prevalence of VI was recorded in 2023 and may be attributed to the sustained efforts at improving access to eye care in Rwanda. However, more data would be needed to confirm this assertion, as other unstudied factors may have influenced this decline. We therefore recommend more research to understand this decrease.

Diseases of the lens were the leading cause of VI in this study. Across the study period, diseases of the lens accounted for more than 50% of all causes of VI. This finding is consistent with other hospital-based studies that reported cataracts as the leading cause of VI (35, 36), but differs from others who found uncorrected refractive errors to be the leading cause of VI (37, 38). The high prevalence of diseases of the lens (cataracts) in this study could be due to the majority of participants belonging to the adult (36–69 years) and elderly (≥60) groups. This is not surprising because increasing age is associated with weakening of bodily cells and a reduction in physiological functions (9, 13, 37). It is believed that the apparent decrease in visual function that manifests earlier in persons with cataract may have contributed to the higher presentation of cataracts than other eye diseases (39). Therefore, sustained efforts are necessary to address the unmet need for addressing cataract prevalence in Rwanda. The Rwanda International Institute of Ophthalmology (RIIO) and the University of Rwanda have started residency programs in Ophthalmology to address the ophthalmology-patient ratio (31). Of note is the magnitude of uncorrected refractive errors in this study. Even though this study found refractive errors as the second leading cause of VI (ranging between 2.6 and 6.4%), the severity was lower than that reported in previous studies in South Africa (38.0%) (38), (28.1%) (37), Nigeria (21.4%) (40), Ghana (19.7%) (35), and Ethiopia (16.1%) (34). Aside from the study design that may have contributed to the lower prevalence of refractive errors in this study, it is speculated that this may be a positive result of the collaboration between the Ministry of Health, Rwanda, and ONESIGHT. Since 2015, the Essilor Luxottica Foundation (through ONESIGHT) has partnered with the Ministry of Health in Rwanda to establish vision centers nationwide, providing affordable spectacles to the population (30–32). This initiative may have contributed to the reduced burden of uncorrected refractive error noted in this study. This study found a higher prevalence of diseases that cause VI among adults and the elderly. This is concerning because the Rwandan population is predominantly youthful and will age in the coming years. As a result, the burden of VI is likely to increase in the general population if adequate actions are not taken to address the existing causes of the disease.

Age was significantly associated (*p* < 0.001) with an increased risk of VI, which ties in with the finding that the risk of VI is significantly higher with diseases of the lens (especially cataracts). These findings align with previous studies, which have shown a significant association between cataracts and a high odds of VI (35, 36). It is to be expected that diseases of the lens, particularly when left untreated, pose a significant risk to the development of VI. Studies have noted that cataracts are prevalent among the elderly due to metabolic changes that occur with advancing age (9, 13). As such, with the surge of older patients presenting at hospitals, it is not unexpected to find significantly high numbers of cataracts that cause VI. With increasing life expectancy and a shift in global demographics towards old age, there is thus a need for conscious efforts to address VI. Strategies to expand cataract surgical coverage and integrate community-based refractive services are urgently needed. Policies that relate to expanding the eye care personnel workforce by training more ophthalmologists, optometrists, opticians, and ophthalmic nurses are essential. This will, among others, improve cataract surgery rates and address the services for refractive error, the two leading causes of VI, consequently reducing the burden of VI. In addition, providing scholarships for specialist training, coupled with post-training bonding, will help address critical staff shortages at public hospitals and ensure the provision of necessary services.

An interesting observation was made regarding gender. The prevalence of VI was higher in females across all study years, in terms of the absolute number of VI cases. This could be attributed to a higher female than male hospital attendance during the study period. However, the risk of developing VI was higher in males than in females. Some authors have tried to explain this paradox. Some schools of thought believe this is because men are involved in more hazardous activities that increases their risk of trauma and, subsequently, VI (41). Others believe that males have a laissez-faire attitude towards their health. It is believed that men will refuse to wear protective equipment, such as goggles, during work and will also downplay their symptoms, thereby not seeking early healthcare when they need it (42). Such activities may contribute to an increased risk of VI among males than females.

### Strengths and limitations of the study

This study has highlighted the prevalence of VI in the general population in Rwanda, emphasizing the trend in VI presentation. It has also highlighted some efforts currently underway in Rwanda to address the prevalence of VI. To the best of our knowledge, a literature search shows that this is the first study to report the prevalence of VI in the general population. Furthermore, this study has a large sample size, includes 6 years of data, and classified VI using ICD-11. However, the study design may limit the interpretation of the results. Hospital-based studies may be biased, as most patients seeking healthcare often require attention, resulting in a higher prevalence of VI being reported. Therefore, the prevalence of VI should be generalized with caution. Additionally, differences in record-keeping at the various hospitals have the potential to affect the quality of data that can be retrieved for such studies. However, this study provides valuable data that can be used to plan eye care services in the community.

## Conclusion

The prevalence of VI in the general population in selected public hospitals ranged between 8.1 and 18.2%. The leading causes of VI were diseases of the lens (cataract), diseases affecting the normal globe (including refractive errors), and diseases of the optic nerve. The leading causes of VI were mainly treatable. Although Rwanda has already implemented some policies to address the burden of VI, further efforts are required to effectively manage the rising burden of VI.

## Data Availability

The data analyzed in this study is subject to the following licenses/restrictions: The data used in this study are available from the public hospitals involved in this study and the Ministry of Health, Rwanda. Requests to access these datasets should be directed to info@moh.gov.rw.

## Appendix 1

**Table:**

Category	Visual acuity worse than	Visual acuity equal to or better than
No vision impairment		6/12
		5/10 (0.5)
		20/40
		0.3
Mild vision impairment	6/12	6/18
	5/10 (0.5)	3/10 (0.3)
	20/40	20/70
	0.3	0.5
Moderate vision impairment	6/18	6/60
	3/10 (0.3)	1/10 (0.1)
	20/70	20/200
	0.5	1.0
Severe vision impairment	6/60	3/60
	1/10 (0.1)	1/20 (0.005)
	20/200	20/400
	1.0	1.3
Blindness	3/60	1/60 (CF at 1 metre)
	1/20 (0.005)	1/50 (0.02)
	20/400	20/1200 (CF at 1 metre)
	1.3	1.8
Blindness	1/60 (CF at 1 metre)	Light perception
	1/50 (0.02)	
	20/1200 (CF at 1 metre)	
	1.8	
Blindness	No light perception	
